# Tumor necrosis factor-α promotes the expression of excitatory amino-acid transporter 2 in astrocytes: Optimal concentration and incubation time

**DOI:** 10.3892/etm.2014.2024

**Published:** 2014-10-15

**Authors:** YUEMIN DING, KENA ZHANG, SHUQIN LIU, QIJUN ZHANG, CHIYUAN MA, IAIN C. BRUCE, XIONG ZHANG

**Affiliations:** 1 School of Medicine, Zhejiang University City College, Hangzhou, Zhejiang 310015, P.R. China; 2 Department of Basic Medicine, College of Medicine, Zhejiang University, Hangzhou, Zhejiang 310058, P.R. China; 3 College of Basic Medical Science, Zhejiang Chinese Medical University, Hangzhou, Zhejiang 310053, P.R. China; 4 Department of Pharmacy, Wannan Medical College, Wuhu, Anhui 241002, P.R. China

**Keywords:** tumor necrosis factor-α, excitatory amino-acid transporter 2, astrocyte, ischemia

## Abstract

The aim of the present study was to determine whether tumor necrosis factor (TNF)-α regulates the expression levels of excitatory amino-acid transporters (EAATs) in primary astrocytes and its roles in brain ischemia. Exogenous TNF-α was administered to pure cultured astrocytes and the expression levels of EAAT1, EAAT2 and glial fibrillary acidic protein (GFAP) were evaluated. The results showed that TNF-α at 10 ng/ml enhanced the expression of EAAT2 in a time-dependent manner, while the expression levels of EAAT1 and GFAP did not change. To determine whether the elevation in the levels of the EAAT2 protein induced by TNF-α had a beneficial effect on ischemic insult, TNF-α was applied to *in vitro* models of cerebral ischemia; the treatment was observed to increase neuronal viability. The present results suggest that a relatively short-term application of an optimal concentration of TNF-α may protect neurons against ischemic injury by elevating the expression of EAAT2 in astrocytes.

## Introduction

Acute ischemic stroke is one of the leading causes of adult disability (1). The excitatory neuronal toxicity caused by the accelerated release of glutamate during brain ischemia is the key element leading to neuronal death. The concentration of glutamate in the synaptic cleft is regulated by membrane-bound excitatory amino-acid transporters (EAATs) (1). To date, five subtypes have been identified and termed EAAT1-5 in human tissues. Glutamate/aspartate transporter, glutamate transporter-1 and excitatory amino-acid carrier 1 are the rodent homologs of EAAT1, 2 and 3, respectively (3). EAAT2 is primarily an astrocytic transporter and is highly expressed throughout the central nervous system (CNS), removing >90% of the total extracellular glutamate from the synaptic cleft (4).

Tumor necrosis factor-α (TNF-α) is an important inflammatory factor and its expression levels markedly increase in the brain following ischemia (5). For a long period of time, TNF-α was believed to induce neuronal necrosis, resulting in brain injury (6), until Cheng *et al* (7) demonstrated that TNF-α (1–100 ng/ml) pretreatment protected cultured neurons from glucose deprivation-induced injury and excitatory amino-acid toxicity. By knocking down the receptors for TNF-α in mice, Gary *et al* (8) unexpectedly observed more severe neuronal damage caused by focal brain ischemia, leading to the hypothesis that TNF-α exhibits a protective role on ischemic neurons. Since then, increasing evidence has shown that TNF-α plays a dual role in ischemic neuronal injury by inducing necrosis and protecting injured neurons (9–12). Our previous study demonstrated that the permeability of the blood-brain barrier to TNF-α transiently increased following injury to the CNS (13). Notably, the time-course of the TNF-α permeability was in accordance with that of the functional recovery, which indicated that the optimal concentration and timing of TNF-α administration contribute to neuroprotection. Although different mechanisms have been proposed to explain the dual role of TNF-α (10–12), the association between TNF-α and EAATs on astrocytes remains unclear. The aim of the present study was to determine whether TNF-α regulates the expression level of EAAT2 in primary astrocytes in culture and its role in brain ischemia.

## Materials and methods

### Animals and reagents

Adult male Sprague Dawley rats were obtained from the Experimental Animal Centre of Zhejiang University (Hangzhou, China). The experimental procedures were approved by the Animal Ethics Committee of Zhejiang University and were carried out in accordance with institutional guidelines. Recombinant rat TNF-α was obtained from BioVision (Milpitas, CA, USA).

### Primary hippocampal neuron culture

Rat hippocampal neurons were prepared as previously described, with certain modifications (14). Briefly, the hippocampi of E18 embryos were dissected and dissociated in oxygenated Hank’s balanced salt solution. Cells (1×10^6^) were plated on coverslips coated with poly-D-lysine (50 μg/ml; Sigma-Aldrich, St. Louis, MO, USA) or on coverslips in Neurobasal^®^ medium supplemented with 2% B27 (Invitrogen Life Technologies, San Diego, CA, USA) and 0.2 mM L-glutamine (Sigma-Aldrich_. Cytosine-D-arabinoside (10 μM; Sigma-Aldrich) was added to the cultures two days after plating to block the proliferation of non-neuronal cells. One-half of the culture medium was changed every four days. After culturing for 12 days, neurons were then co-cultured with astrocytes.

### Primary cortical astrocyte culture and TNF-α treatment

Astrocytes in primary culture were prepared from the cerebral cortices of newborn (0–12 h postnatal) rat pups as previously described (15). In brief, cells were trypsinized with 0.25% trypsin (Invitrogen Life Technologies, Carlsbad, CA, USA) for 16 min, followed by trituration with 10 mg/l DNAse (Sigma-Aldrich). Cells were plated in poly-D-lysine-coated flasks and maintained in Dulbecco’s Modified Eagle’s medium (DMEM; Invitrogen Life Technologies) supplemented with 10% fetal bovine serum (PAA, Pasching, Austria). The culture medium was changed every third day. After nine days, the flasks were agitated for 12 h at 37°C to separate the microglia from the more adherent mass of astrocytes. The adherent cells were replated in dishes and cultured for another week prior to experimentation. Immunostaining with the glial fibrillary acidic protein (GFAP) antibody (Cell Signaling Technology, Inc., Danvers, MA, USA) confirmed that the majority (>95%) of the cells expressed GFAP (data not shown). To examine the dose-dependent effect, recombinant rat TNF-α (1, 10, 20 and 50 ng/ml) was added directly to the medium and the cells were lysed following 24-h incubation. To explore the time-dependent effect, astrocytes were treated with 10 ng/ml TNFα for 4, 8, 12, 24, 36 or 72 h and lysed after treatment.

### Co-culture of astrocytes and neurons

Co-cultures of astrocytes and neurons were prepared as previously described, with certain modifications (16). Briefly, astrocytes in Petri dishes were washed twice with phosphate-buffered saline (PBS) and incubated in serum-free medium for 3 h. Glass coverslips with hippocampal neurons were inverted over the separately-cultured astrocytes so that the neurons were facing the astrocytes. The cells were incubated together in Neurobasal medium containing B27 and L-glutamine for 48 h to induce direct contact. The cultures were pretreated with 10 ng/ml TNF-α for 0–24 h prior to being subjected to oxygen-glucose deprivation (OGD).

### ODG

OGD was performed as previously described (17). Briefly, co-cultured cells were washed with PBS and incubated in glucose-free DMEM. The cultures were transferred to an anaerobic chamber filled with a gas mixture of 95% N_2_ and 5% CO_2_ at 37°C for 2 h. Exposure was terminated by removing the glucose-free DMEM and adding Neurobasal medium. After 22 h of recovery in a normoxic incubator, the coverslips were carefully transferred, face-up, to a new 24-well plate containing Neurobasal medium and the cell viability was assessed as described below. In each experiment, cultures exposed to OGD were compared with normoxic controls supplied with DMEM containing glucose and maintained in standard incubation conditions.

### MTT assay

Cell viability was measured using the MTT (Sigma-Aldrich) assay. Cells were gently washed with fresh culture medium, and DMEM and the serum-free medium containing MTT (5 mg/ml) was added and incubated for an additional 4 h at 37°C. The purple-blue MTT formazan precipitate was dissolved in 150 μl dimethyl sulfoxide. Absorbance was measured at 490 nm.

### TNF-α treatment in vivo

A total of 6 male rats were anesthetized by an intraperitoneal injection of 2% sodium pentobarbital (40 mg/kg) and mounted in a stereotaxic frame on a heating blanket. TNF-α was administered according to previously described techniques (18), with certain modifications. Intracerebroventricular (i.c.v.) injection was performed at the following coordinates: 0.6 mm posterior, 4.5 mm ventral and 1.6 mm lateral to the bregma. Recombinant rat TNF-α (100 ng/15 μl) was injected into the left lateral ventricle over a period of 10 min. A total of 3 rats in the control group received the same volume of sterilized, artificial cerebrospinal fluid. The expression levels of the EAATs in the cerebral cortex were determined by western blot analysis 24 h after treatment with TNF-α.

### Western blot assay

Cerebral cortical tissues or astrocytes were lysed on ice in radioimmunoprecipitation assay buffer with a protease inhibitor cocktail (Sigma-Aldrich) containing a mixture of several protease inhibitors, including AEBSF at 104 mM, Aprotinin at 80 μM, Bestatin at 4 mM, E-64 at 1.4 mM, Leupeptin at 2 mM and Pepstatin A at 1.5 mM. Protein content was estimated using the bicinchoninic acid assay method. Immunoblotting was performed as described previously (19). Following electrophoresis, protein samples were transferred to polyvinylidene fluoride membranes. The membranes were incubated at 4°C overnight with antibodies against EAAT1 (1:500; Wuhan Boster Biological Technology, Ltd., Wuhan, China), EAAT2 (1:500; Wuhan Boster Biological Technology, Ltd.), GFAP (1:2,000) or β-actin (1:1,000; Cell Signaling Technology, Inc.) diluted in blocking solution. Following 2 h of incubation with a horseradish peroxidase-labeled secondary antibody (Jackson ImmunoResearch Laboratories, West Grove, PA, USA) at room temperature, the blots were developed with enhanced chemiluminescence reagents (Pierce, Rockford, IL, USA) and exposed to X-ray film to obtain images. The intensity of each band was quantified using ImageJ software (National Institutes of Health, Bethesda, MD, USA) and normalized to β-actin.

### Statistical analysis

Data are expressed as the mean ± standard deviation. Comparisons between groups were analyzed using the Student’s t-test or one-way analysis of variance, followed by Dunnett’s post hoc test. P<0.05 was considered to indicate a statistically significant difference.

## Results

### TNF-α elevates EAAT2 expression in vivo

**Figure 1:**
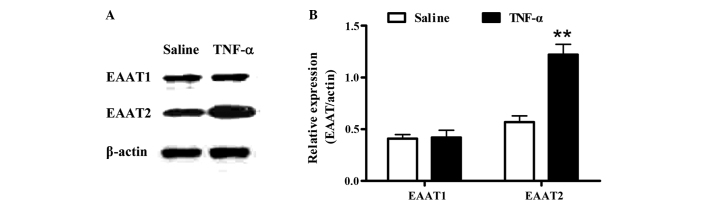
TNF-α treatment elevates the expression of EAAT2 in the brain. (A) Expression levels of the EAATs following TNF-α treatment. (B) Quantification of the expression levels of the EAATs. Data are presented as the mean ± standard deviation and were analyzed by one-way analysis of variance (n=3). ^**^P<0.01 compared with saline controls. TNF-α, tumor necrosis factor-α; EAAT, excitatory amino-acid transporter.

To investigate the effect of TNF-α on the expression of EAATs *in vivo*, recombinant rat TNF-α was administered by i.c.v. injection and the expression levels of the EAATs in the cerebral cortical tissue were assessed by western blot analysis. Compared with the EAAT2 levels in the saline control group, EAAT2 expression markedly increased (P<0.01) after TNF-α treatment for 24 h. However, no changes were observed in the expression level of EAAT1 (Fig. 1).

### TNF-α regulates EAAT2 expression in astrocytes

**Figure 2:**
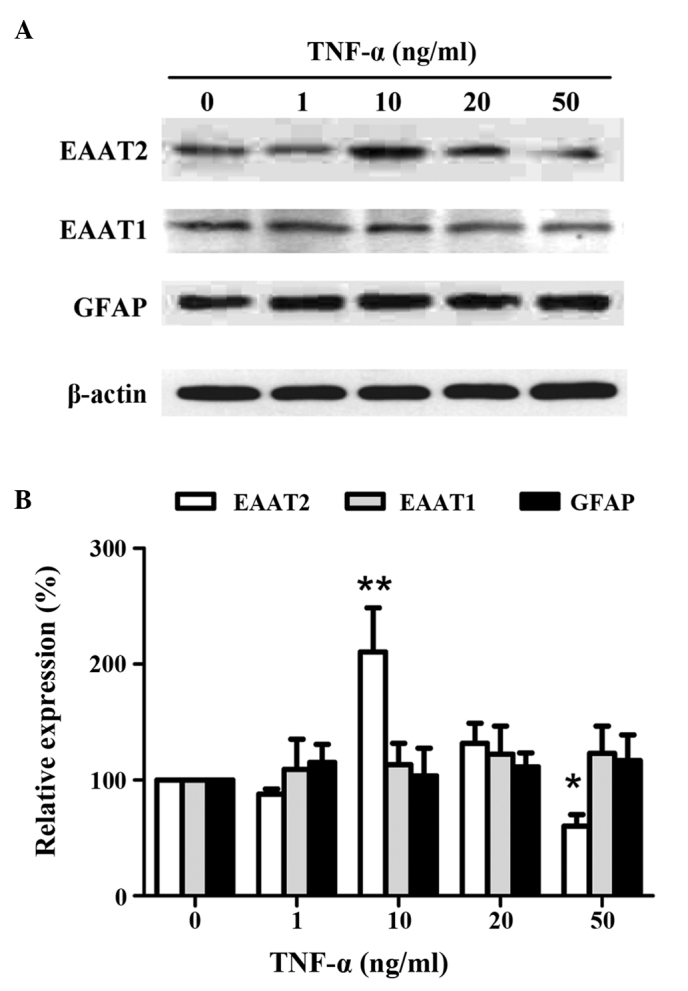
Expression of EAATs in astrocytes treated with different doses of TNF-α. (A) Representative western blot analysis of EAAT1, EAAT2, GFAP and β-actin. (B) Densitometric analysis showing the expression of EAATs or GFAP normalized to β-actin. Data are presented as the mean ± standard deviation (n=5). ^*^P<0.05 and ^**^P<0.01 compared with the control group (0 ng/ml TNF-α). TNF-α, tumor necrosis factor-α; EAAT, excitatory amino-acid transporter; GFAP, glial fibrillary acidic protein.

To determine whether the expression levels of EAAT proteins in astrocytes are regulated by TNF-α, the dose-dependent effect of TNF-α (1, 10, 20 or 50 ng/ml) on primary cortical astrocytes was analyzed (Fig. 2). The optimal concentration of TNF-α (10 ng/ml) upregulated EAAT2 expression (P<0.01), whereas a high concentration of TNF-α (50 ng/ml) negatively regulated EAAT2 expression (P<0.05). Different doses of TNF-α did not change the expression levels of EAAT1 or GFAP, the latter representing astrocyte proliferation (Fig. 2).

**Figure 3:**
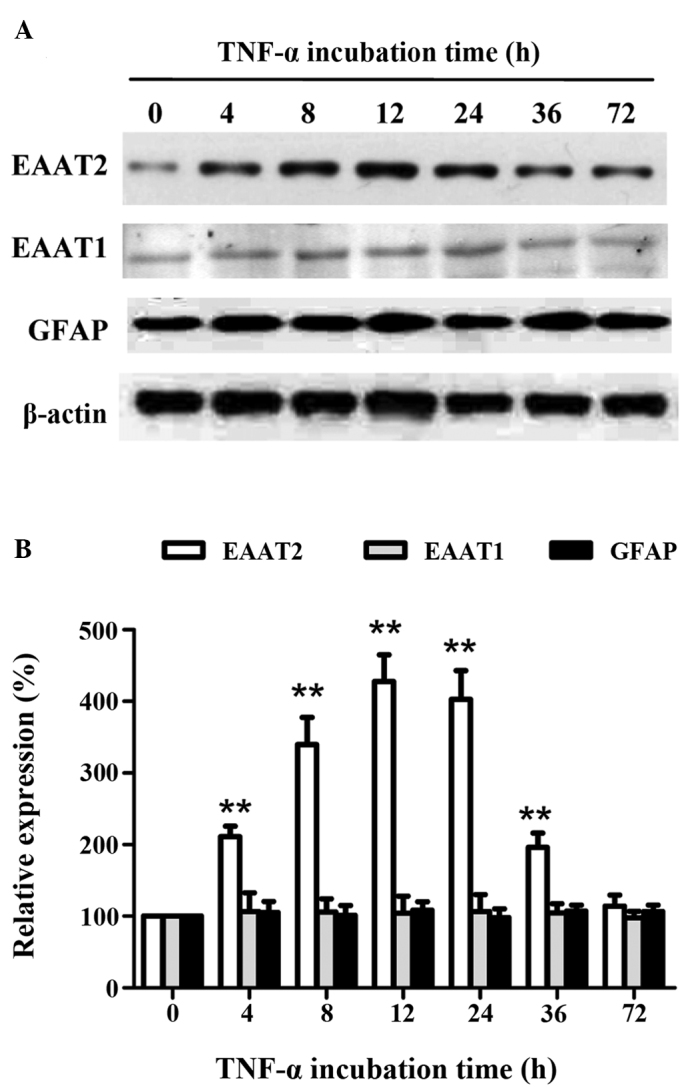
Expression levels of EAATs in astrocytes treated with TNF-α for different time-periods. (A) Representative western blot analysis of EAAT1, EAAT2, GFAP and β-actin. (B) Densitometric analysis showing the expression levels of the EAATs or GFAP normalized to β-actin. Data are presented as the mean ± standard deviation (n=6). ^**^P<0.01 compared with the control group (prior to TNF-α treatment, 0 h). TNF-α, tumor necrosis factor-α; EAAT, excitatory amino-acid transporter; GFAP, glial fibrillary acidic protein.

To further explore the association between incubation time and the expression levels of EAATs in astrocytes, the astrocytes were treated with the optimal concentration of TNF-α (10 ng/ml) for different incubation periods (4, 8, 12, 24, 36 or 72 h). The EAAT2 protein expression showed a time-dependent increase, followed by a time-dependent decrease, with a maximum level (P<0.01) after 12 h (Fig. 3). However, the expression levels of EAAT1 and GFAP did not change with treatment. These results indicated that short-term treatment with the optimal concentration of TNF-α promoted EAAT2 expression in astrocytes, which was independent of astrocyte proliferation.

### TNF-α treatment increases neuronal viability following OGD injury

**Figure 4:**
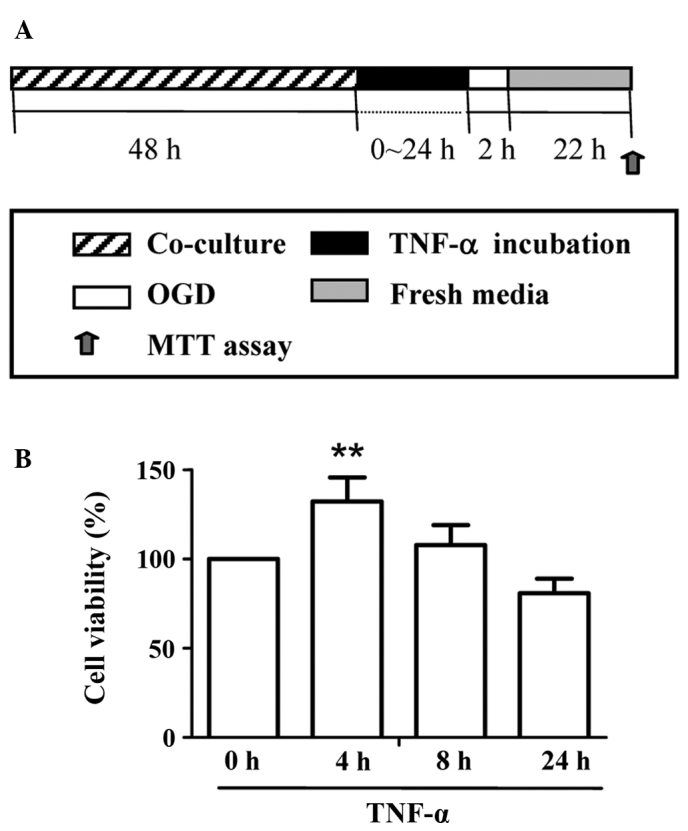
Effect of TNF-α pretreatment on neuronal viability following OGD. (A) Timeline of the experimental procedure. At nine days *in vitro* the neuron and astrocyte co-cultures were set up and maintained for 48 h. The cultures were pretreated with 10 ng/ml TNF-α for 0–24 h prior to OGD and subsequently placed in fresh medium for 22 h to recover. Neuronal viability was measured by MTT assay. (B) MTT results showing that short-term (4 h) TNF-α pretreatment had a distinct neuroprotective effect against OGD injury. Data are presented as the mean ± standard deviation (n=5). ^**^P<0.01 compared with the corresponding 0 h group. TNF-α, tumor necrosis factor-α; OGD, oxygen-glucose deprivation.

To determine whether TNF-α treatment is neuroprotective against neuronal ischemic injury *in vitro*, a co-culture system of astrocytes and neurons was established and cell viability was assessed following OGD injury. After different periods of incubation (0–24 h) with 10 ng/ml TNF-α, OGD was applied for 2 h, followed by 22 h of reoxygenation (Fig. 4A). An MTT assay was performed on the hippocampal neurons to reveal the cell viability following OGD insult. The results demonstrated that only short-term (4 h) TNF-α treatment led to a significantly higher cell viability value compared with the other groups (P<0.01) (Fig. 4B).

## Discussion

Clinical studies have demonstrated that a transient ischemic attack (TIA) within a narrow time-window may enhance the tolerance of the brain against a more severe ischemic insult (20–22). Notably, elevation in the plasma levels of TNF-α have been reported in patients for <72 h following a TIA (23), leading to the proposal that TNF-α pretreatment may mimic the neuroprotective effect of ischemic preconditioning. Coinciding with this hypothesis, it has been found that the elevation in TNF-α levels induced by ischemic preconditioning and direct TNF-α pretreatment contributes to the enhancement of cellular defense against the second insult (10,15,24). In the present study, *in vivo* TNF-α treatment markedly decreased the infarct volume in the ischemic brain and improved the functional recovery of rats receiving a subsequent middle cerebral artery occlusion (data not shown).

Numerous studies have been performed to investigate the mechanisms of ischemic tolerance caused by TNF-α exposure. Glazner and Mattson found that TNF-α pretreatment reduced calcium influx and upregulated the gene transcription of neuroprotective factors (25). In addition, Watters and O’Conner (26) and Watters *et al* (27) revealed that TNF-α pretreatment increased the resistance of neurons to a subsequent insult from glutamate by attenuating resting calcium activity and calcium-related responsiveness. In the present study, the expression level of EAAT2, but not EAAT1, was upregulated by TNF-α treatment *in vivo* and *in vitro*. This was consistent with the findings of Davis and Patel (28), who demonstrated that the expression level of EAAT2 was significantly increased in ischemic preconditioning, suggesting that EAAT2 upregulation attenuates the toxicity of the excitatory glutamate induced by a more severe ischemic insult. This was further confirmed by the study of Weller *et al* (29), in which it was observed that astrocytes that had undergone transfection to overexpress EAAT2 played a marked protective role against moderate ischemia.

Consistent with the present results, Tilleux *et al* (30) reported the stimulatory effects of TNF-α on EAAT2 expression, although other studies have found inhibitory effects. Su *et al* (31) revealed that the mRNA expression of EAAT2 began to decline from 48 h with TNF-α (30 ng/ml) treatment. Thus, the effect of TNF-α on EAAT2 expression remains controversial. In the present study, TNF-α treatment promoted the expression of EAAT2 in astrocytes in a concentration- and time-dependent manner. The current results suggested that a lower concentration (<20 ng/ml) of TNF-α applied as a short-term (<36 h) treatment contributed to the induction of a neuroprotective effect by elevating EAAT2 expression, while this effect was lost or even reversed with increasing concentration and incubation time. Although the underlying mechanism remains unclear, this may explain why certain studies provide evidence for an inhibitory influence of TNF-α on EAAT2 expression. Furthermore, it was demonstrated in the present study that incubation with TNF-α did not increase astrocyte proliferation. Therefore, the elevated expression of EAAT2 in astrocytes was proliferation-independent.

To further determine whether the elevated levels of EAAT2 protein induced by TNF-α had a beneficial outcome *in vitro*, the viability following OGD injury in a co-culture system of astrocytes and neurons was assessed. Corresponding with the *in vivo* results, it was revealed that TNF-α increased the cell viability following OGD injury, suggesting that it has a neuroprotective effect by promoting EAAT2 expression in astrocytes.

The present study had several limitations. Firstly, based on previous findings, TNF-α may have dual effects on the expression and activity of EAATs. Thus, detection of the activity of EAATs under these experimental conditions is required. Secondly, it is necessary to demonstrate whether the protection is eradicated following the blockade of glutamate uptake. Finally, an advanced mechanistic study is also required. Therefore, these issues should be addressed in future studies.

In conclusion, the present study revealed that an optimal concentration and time-course of TNF-α elevates EAAT2 expression in astrocytes and has a beneficial effect on subsequent ischemic insult.
